# Global research progress and trends in traditional Chinese medicine for chronic kidney disease since the 21st century: a bibliometric analysis

**DOI:** 10.3389/fmed.2024.1480832

**Published:** 2025-01-17

**Authors:** Heyong Wang, Jun Wang, Yang Chen, Dianxing Yang, Lanyue Xiong

**Affiliations:** ^1^School of Basic Medical Sciences, Chengdu University of Traditional Chinese Medicine, Chengdu, China; ^2^Department of Nephrology, Sichuan Integrative Medicine Hospital, Chengdu, China; ^3^School Clinic, Luoyang Vocational College of Culture and Tourism, Luoyang, Henan Province, China; ^4^Department of Cardiovascular, Chengdu First People's Hospital, Chengdu, China

**Keywords:** chronic kidney disease, traditional Chinese medicine, oxidative stress, network pharmacology, gut microbiota

## Abstract

**Objective:**

This study analyzed literature on traditional Chinese medicine (TCM) in treating chronic kidney disease (CKD) to identify research trends and provide guidance for future studies and clinical practice.

**Methods:**

The study used data from Web of Science from 2000 to 2024 to analyze English-language literature on CKD and TCM. Bibliometric analysis was done using R software and the bibliometric package, with scientific mapping and visualization analysis conducted using tools like Citespace, VOSviewer, and ScimagoGraphica to explore research trends and connections.

**Results:**

This study revealed that a total of 1,153 relevant documents were retrieved, and the number of published articles showed an increasing trend, reaching a peak in 2022. In terms of article publication, China ranked first with 760 articles, closely followed by the United States with 132 articles. Guangzhou University of Traditional Chinese Medicine published 60 papers, the most among academic institutions, followed by Shanghai University of Traditional Chinese Medicine with 54 papers. In terms of individual authors, Liu Xinhui holds the record for the highest number of published articles, totaling 17, followed by Li Ping and Li Shunmin. The prevalent keywords include “chronic kidney disease,” “TCM,” and “oxidative stress.” Currently, the prominent areas of research interest include network pharmacology, gut microbiota, oxidative stress, and related topics. The current research trend in this field is towards the adoption of novel methodologies such as network pharmacology and the emphasis on exploring the relationship between gut microbiota and CKD.

**Conclusion:**

Global research on TCM in the treatment of CKD is showing a positive development trend, but further research on safety, efficacy evaluation, and international cooperation is still needed. The development trend is to adopt new scientific research methods and focus on exploring the mechanism of TCM in treating CKD.

## 1 Introduction

Chronic Kidney Disease (CKD) represents a significant and growing global public health concern, affecting the health of 800 million people worldwide (1). The complex pathophysiology of CKD is progressive kidney damage that leads to a gradual decline in kidney function over time and may eventually develop into end-stage renal disease (ESRD) (2). The treatment of CKD has been significantly enhanced by diverse medical progressions, encompassing targeted cytokines, transcription factors, developmental and signaling pathways, and epigenetic regulators, especially microRNAs. For example, the FXR agonist GW4064 mitigated glomerulosclerosis and interstitial fibrosis through suppressing SREBP1 expression (3). The selective FXR agonist INT-747 modulated lipid accumulation, perhaps by inhibiting NF-κB transcriptional activity, and improved proteinuria, glomerulosclerosis, and tubulointerstitial fibrosis in a streptozotocin-induced diabetic nephropathy mouse model (3). Additionally, the activation of transcription factor Nrf2 was associated with CKD regression, indicating its potential as a drug target (4). MicroRNA expression alterations in CKD are closely tied to its development, and their roles in various CKD states such as diabetic nephropathy and lupus nephritis have been widely explored (5, 6). Despite these advancements, CKD shows a gradual progression and ultimately leads to irreversible nephron loss, ESRD, and premature mortality (7). CKD is a major global cause of death, ranking 16th worldwide and claiming millions of lives each year (8, 9). The economic burden of CKD is increasing due to an aging population and growing incidence of comorbid diseases (10). CKD has a significant economic impact on healthcare, with costs rising by 1.3 to 4.2 times when progressing from stage 3 to stage 4 or 5, and an estimated annual fee of $20,000–100,000 per patient for ESRD progression (11). As a result, there is an urgent need to identify novel therapeutic strategies for the treatment of CKD.

TCM has been widely used to treat CKD in China and other countries for centuries. TCM uses herbal medicines, acupuncture, and other complementary therapies to treat various diseases, including CKD (12–14). Renal fibrosis is a common feature of CKD progression. Currently, there is no ideal treatment method. In recent years, clinical evidence and experimental studies have shown that based on TCM CKD theory and molecular mechanism, various traditional Chinese medicines and their active ingredients can delay the progression of CKD and have multi-target and multi-functional characteristics (15). The scientific evidence regarding the effectiveness and safety of TCM in managing CKD is inconclusive, with varying results reported in different studies. Additionally, concerns about the potential nephrotoxic effects of TCM have sparked debate regarding its role in CKD treatment (16, 17).

Bibliometric analysis is a quantitative approach to evaluating scientific output, and it can provide insights into research trends, hotspots, and influential authors in a particular field (18). For instance, a bibliometric analysis was conducted to examine the worldwide research patterns and focal points concerning autophagy and kidney disease between 2000 and 2022 (19), while another study focused on Emerging trends and focus for the link between the gastrointestinal microbiome and kidney disease (20). Zhang et al. performed a bibliometric analysis of citation classics on clinical intervention trials of exercise training for CKD, and depicted the research landscape and hotspots in this field (21). However, to date, there is only one bibliometric study on the research and application of Chinese herbal medicine in the treatment of CKD (22), and no bibliometric analysis on the treatment of CKD with TCM has been published yet.

In response to address the research gap, this study conducts a comprehensive bibliometric analysis of the literature on TCM for the treatment of CKD from 2000 to 2024, using the Web of Science database. The analysis addresses several key questions to guide future research and clinical practice. Specifically, the study investigates the current research trends and key areas of focus in TCM for CKD treatment. It also identifies the most influential authors, institutions, and countries contributing to this field. Additionally, this study explores emerging themes and methodologies that are shaping the research landscape. Finally, it aims to uncover gaps in the literature, particularly concerning the efficacy and safety of TCM in treating CKD, and proposes areas where further research is needed. By answering these questions, this study seeks to offer valuable insights for future studies and clinical application.

## 2 Methods

### 2.1 Data sources and search strategies

In this study, we obtained literature data from the Web of Science (WOS). To ensure that the data we received was both adequate and comprehensive, we utilized two essential components of the Web of Science Core Collection (WoSCC): the Science Citation Index Expanded (SCIE) and the Social Science Citation Index (SSCI). WOS is the most renowned and dependable citation index due to its stringent criteria for the evaluation and selection of journals, and its ability to provide valid and reliable information (23). The period of the study was from January 1, 2000 to January 20, 2024, which ensured comprehensive coverage of the literature we searched. During the literature search, we specified the type of literature to include only ARTICLE and REVIEW, while excluding other types of articles such as editorials, conference proceedings, and letters. At the same time, we set the language to English to minimize the potential comprehension bias arising from language differences. This was done to ensure the consistency and reliability of the data for our analysis. Additionally, we further filtered the literature based on specific keywords and topics related to TCM for CKD to ensure the relevance of the included articles. Finally, we used (TS = CKD OR chronic kidney disease OR chronic renal disease OR chronic renal insufficiency OR chronic kidney insufficiency) AND (TS = patent herbal drug OR herbal medication OR Chinese patent medicine OR herbal medicine OR Chinese herbal preparation OR Chinese herbal medicine OR herbal formulas OR herbal extract OR Chinese herbal decoction OR herbal supplement OR Chinese medicine OR herbal products OR traditional Chinese medicine OR traditional medicine) AND (TS = patent herbal drug OR herbal medication OR Chinese patent medicine OR herbal medicine OR Chinese herbal preparation OR Chinese herbal medicine OR herbal medicine OR traditional herbal medicine OR traditional medicine) as the final search strategy. All search results were exported from WOS and temporarily saved in plain text files for subsequent data processing and further analysis.

### 2.2 Data collection and processing

In the course of the study, we retrieved and downloaded the data retrieved from WOS on January 25, 2024 to avoid possible errors associated with frequent database updates, and we successfully downloaded a total of 1,153 documents that met the screening criteria of the study. In order to better understand the characteristics and trends of the literature, we adopted R software version 4.2.1 and used “bibliometrixpackage4.0.1” as a tool for bibliometric analysis and data collection. The choice of this tool was based on its wide application and efficiency in the field of literature research. During the data importation process, great attention was paid to the accuracy and completeness of the data. Therefore, after importing the downloaded literature data into the R software, we performed a systematic integrity check and cleaning to ensure the quality of the analyzed data. Subsequently, we imported the critical information of each literature into Microsoft Office Excel (2019 version) in an orderly manner and stored it properly.

### 2.3 Bibliometric analysis

We employed the Bibliometrix R software package, CiteSpace, and VOSviewer for different aspects of bibliometric analysis. Each aspect makes a unique contribution to answering research questions. Bibliometrix provides quantitative insights into publication trends, citation analysis, and key author/institution indicators (24), which helps to identify influential research and key players in the field of TCM and CKD. CiteSpace is used to detect research frontiers and knowledge gaps by mapping citation bursts, co-citation analysis, and keyword clustering (25), thereby helping us identify emerging topics and research hotspots. VOSviewer is used for network visualization, including co-authorship networks and national cooperation (26), which illustrates the degree of international cooperation and the structure of scientific influence in this field. This study adopted a combined approach of scientometric analysis and systematic review, similar to the methodology employed by Gholampour et al. (27, 28).

Regarding parameter selection, we chose default settings in some cases, but in other cases, we applied custom parameters to improve the specificity of the analysis. For example, in Bibliometrix, we used a threshold of at least five citations per article to focus on influential works. In CiteSpace, we adjusted the time slice to a two-year interval (2000–2024) to capture the temporal evolution of research trends. For VOSviewer, we selected a minimum co-occurrence threshold of 10 for keywords to ensure that only important trends and connections are visualized. These adjustments were made to optimize the relevance and clarity of the analysis and ensure that the output is directly aligned with our research goals.

## 3 Results

### 3.1 Literature search results

**Figure 1:**
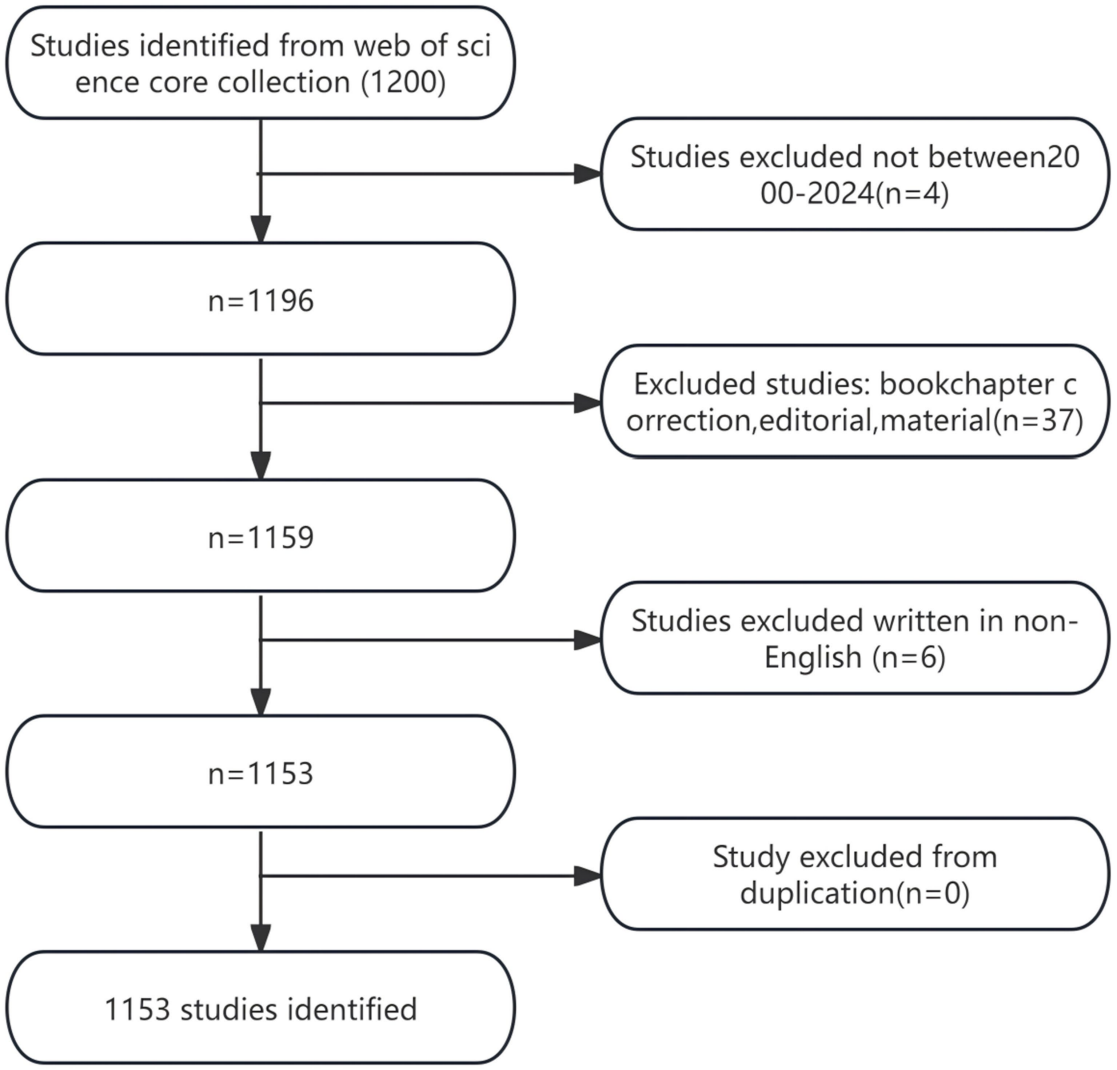
Flow chart of the literature search.

The search yielded a total of 1,200 articles pertaining to the correlation between TCM and CKD in the WOSCC database, spanning from January 1, 2000, to January 20, 2024. After excluding 37 studies categorized as book chapter corrections, editing, or materials, as well as six non-English studies, no duplicate studies were identified, resulting in a final count of 1,153 relevant studies. The details of the literature search process can be found in Figure 1.

### 3.2 Distribution of literature

**Figure 2:**
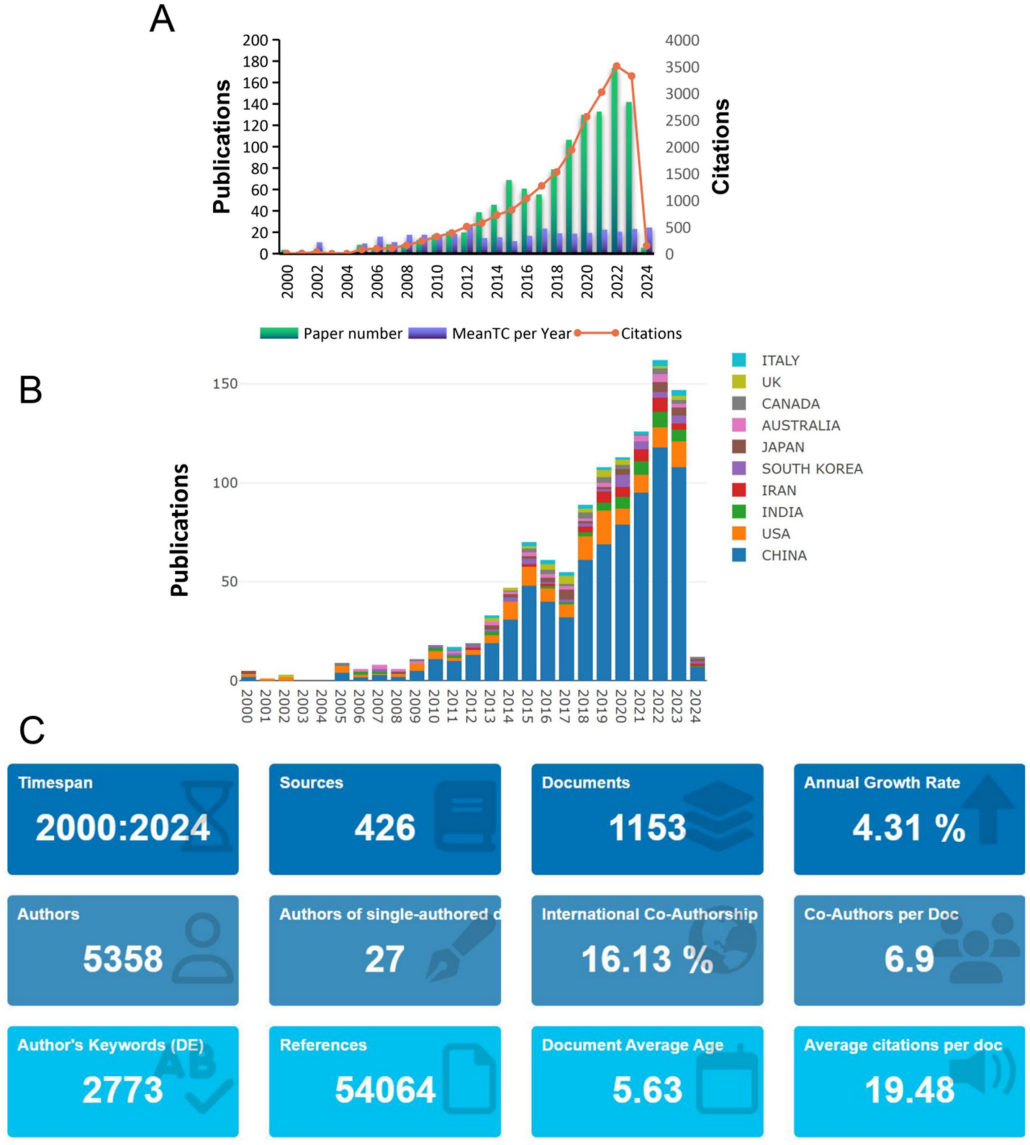
Literature distribution analysis. **(A)** Publication trends from 2000 to 2024, showing a significant increase in recent years, especially in 2022. **(B)** Top 10 countries by publication volume, with China and the U.S. leading. **(C)** General overview of the included literature, reflecting the growing global interest in TCM and CKD research.

Through the search strategy mentioned in the Methods section, we retrieved a total of 1,153 eligible articles (Figure 2A). These articles span from 2000 to 2024, and the number of publications increases year by year, showing three main phases: the first phase is from 2000 to 2005, in which the number of published articles is low and offers a slow growth trend; the second phase is from 2006 to 2017, in which the number of documents starts to increase, and the increase becomes more extensive than before, with the number per year up to more than 50 articles; the third phase is from 2019 to 2024, the number of articles published within these years increased dramatically, indicating that the study became a research hotspot in recent years, and the year with the highest number of published literature is 2022, with the number of all 174 articles, and the citation frequency is at the highest end of the scale, with the total number of citations in the literature being 3,508, which indicates that more high-quality studies were published in this year. The highest average number of literature citations was in 2012, which equates to an average of 25 citations per document in that year. The country with the highest percentage of publications is China, followed by the United States; the distribution of the top 10 countries with the highest number of publications in terms of the year of publication is shown in (Figure 2B), where China has been involved in the publication of articles in the related field from 2000 to 2024, with a very high level of contribution to the field; followed by the United States, where the number of publications has gradually increased in the field from 2008 onwards. According to R software statistics, these 1,630 articles came from 5,358 co-authors from 77 countries, were published in 426 journals, and cited a total of 54,064 references (Figure 2C). The country with the highest percentage of publications is China, followed by the United States. This dominance reflects China’s long-term investment and policy support for TCM research, especially with a focus on chronic diseases like CKD. The United States, despite publishing fewer papers, shows a higher citation per article, which can be attributed to its extensive international collaborations and its leadership in global research funding. Additionally, countries like India, Iran, South Korea, and Japan have been able to establish themselves as scientific leaders in this field due to the increasing governmental support, academic infrastructure, and a growing focus on complementary and alternative medicine.

### 3.3 Analysis of national communications

**Figure 3:**
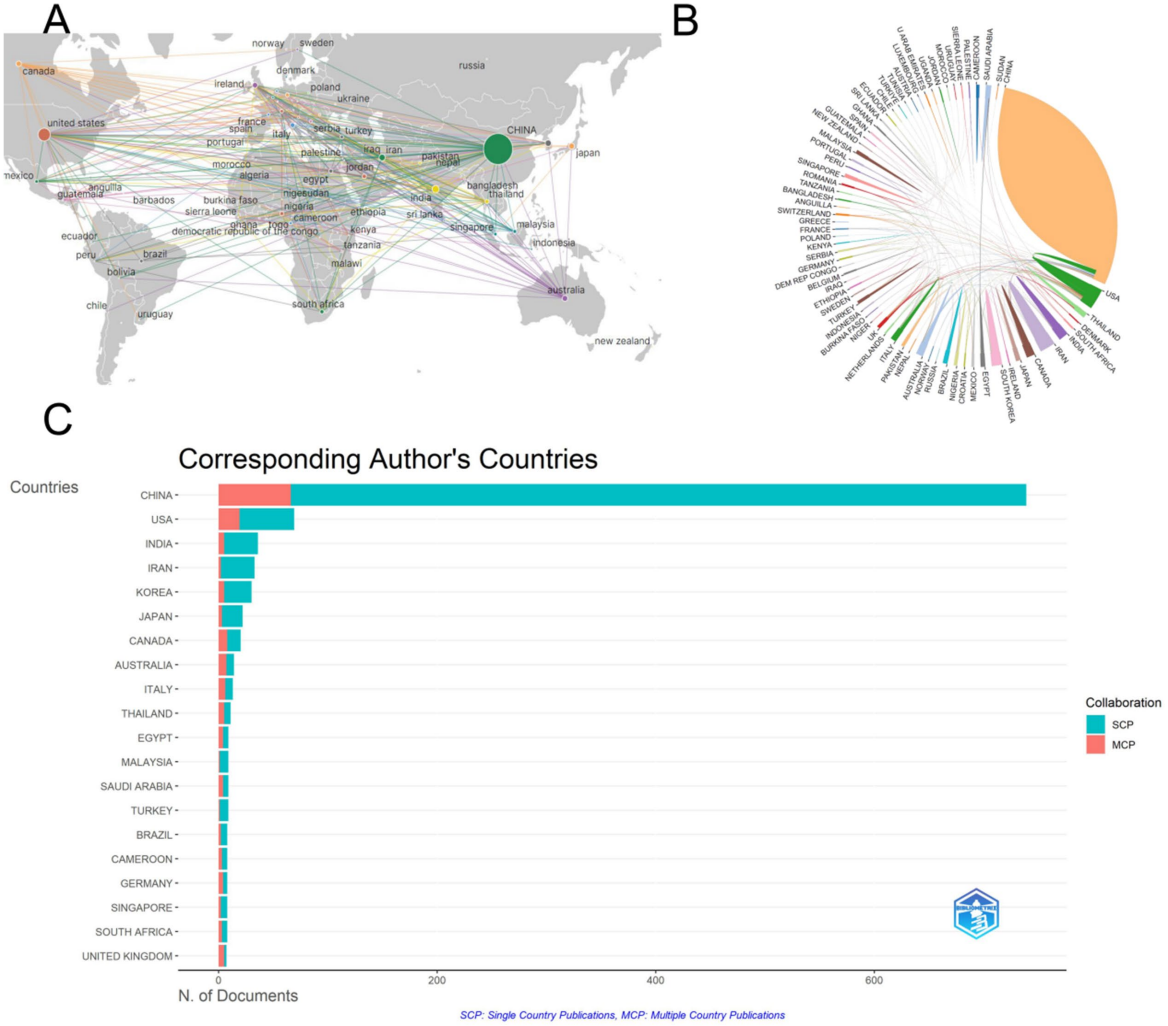
Analysis of country releases for included articles. **(A)** Visualization map of country cooperation, showing that China, the United States, and European countries are the main contributors in this research area. **(B)** Chordal map of country cooperation, indicating that collaborations are mainly centered around China and the United States, with most collaborations involving developed countries. **(C)** Map ranked by the number of articles for country cooperation, revealing that the UK has the highest percentage of international collaborative articles, while some countries have fewer international collaborations.

Among the 1,153 papers included in the study, we conducted a visual network analysis of the countries involved in the issuance of the documents, and the number of statistical countries was not limited to the first and corresponding authors. Still, it was distributed in 77 countries, mainly in China, Europe, and the United States (Figure 3A). China has the highest number of literature with 760, followed by the United States with 132 and India with 44, while the rest of the countries do not have more than 50 literature (Table 1). The countries with the highest number of citations in literature were China with 12,885 citations in published literature, the United States with 6,107 citations in published literature and India with 697 citations in published literature. (Figure 3B) shows the visualized international collaboration network, with the size of the circle and chord plot area representing the number of citations. It can be seen that most of the collaborations are related to China and the United States, mostly centered on developed countries, while collaborations among other countries are relatively weak. Most countries have multinational collaborative research, with the United Kingdom having the highest percentage of international collaborative articles, and IRAN, Japan and Korea having fewer international collaborative articles (Figure 3C). This outcome might reflect China’s long-term scientific research investment and policy support in the field of traditional Chinese medicine, particularly the encouragement of research on traditional Chinese medicine at the national level. On the other hand, the high citation frequency in the United States might be related to its significance in the global scientific research system and the extensiveness of its scientific collaborations.

**Table 1:** Top 10 countries with the most publications.

Rank	Country/region	Article counts	Centrality	Percentage (%)	Citation	Citation per publication
1	China	760	0.25	53.9%	12,885	16.95
2	United States	132	0.36	9.4%	6,107	46.27
3	India	44	0.04	3.1%	697	15.84
4	Iran	34	0.01	2.4%	305	8.97
5	South Korea	33	0.02	2.3%	415	12.58
6	Japan	29	0.08	2.1%	302	10.41
7	Australia	27	0.05	1.9%	824	30.52
8	Canada	25	0.09	1.8%	526	21.04
9	United kingdom	24	0.05	1.7%	937	39.04
10	Italy	19	0.10	1.3%	457	24.05

### 3.4 Organizational analysis

**Figure 4:**
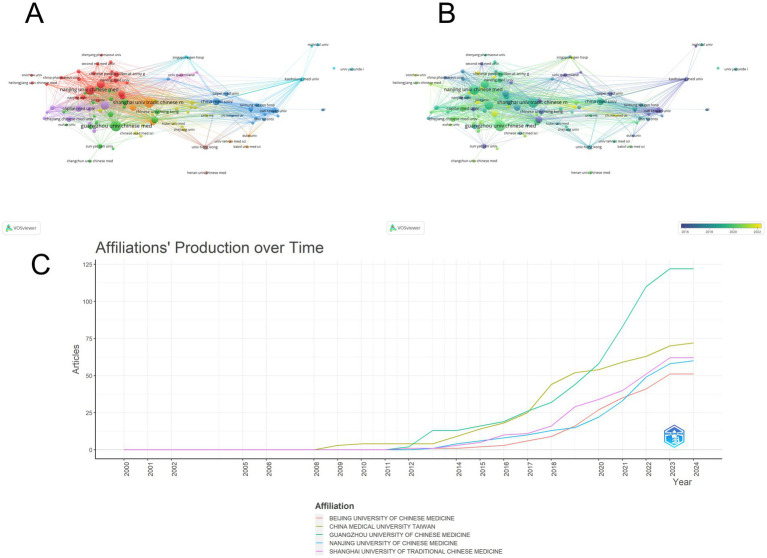
Analysis of the issuing institutions. **(A)** Visualization map showing cooperation between institutions, with more collaborations among Nanjing, Shanghai, and Guangzhou Universities of TCM. **(B)** Time map indicating that the average publication year is centered, and most papers are from the last 10 years. **(C)** Posting patterns of the top five institutions, showing the influence of Guangzhou and Shanghai Universities of TCM and their recent publication activity.

We performed network visualization and density visualization analysis of issuing organizations by VOSViewer software. (Figures 4A,B) The connecting line represents the cooperation relationship between organizations, the size of the circle represents the number of times of issuance, and the node color changes from purple to yellow to represent the passage of time. We set the minimum number of collaborations as 5 times and constructed 102 inter-organizational collaborations. Among them, there were more collaborations with Nanjing University of TCM, Shanghai University of TCM and Guangzhou University of TCM, and most of them were TCM-related medical universities. At the same time, the other organizations maintained a stable pattern of group collaborative research, and there were fewer collaborations between multinational organizations. The average year of literature published by these organizations is more centered, and most of them have published papers in the last 10 years. (Figure 4C) shows the trend of publications for some organizations; institutions are not limited to those to which the first and corresponding authors belong. The largest number of publications was from the Guangzhou University of Traditional Chinese Medicine (60); followed by the Shanghai University of Traditional Chinese Medicine (54) and Nanjing University of Chinese Medicine (49) (Table 2). It can be seen that Guangzhou University and Shanghai University of Traditional Chinese Medicine have a large influence in this field, and the number of articles published in recent years is also very high. In conclusion, the analysis indicates that there is a relatively concentrated collaboration pattern among TCM-related medical universities in China, and these universities have made substantial contributions to the research in this field in recent years. However, there is still room for further enhancing international collaboration to promote the development of TCM research on a global scale.

**Table 2:** Top 10 institutions with the most publications.

Rank	Institution	Country	Number of studies	Total citations	Average citation
1	Guangzhou University of Chinese Medicine	China	60	510	8.50
2	Shanghai University of Traditional Chinese Medicine	China	54	765	14.17
3	Nanjing University of Chinese Medicine	China	49	782	15.96
4	Beijing University Of Chinese Medicine	China	38	572	15.05
5	China academy of Chinese medical sciences	China	34	499	14.68
6	China Medical University	China	29	499	17.21
7	Peking University	China	25	456	18.24
8	Fudan University	China	23	571	24.83
9	Capital Medical University	China	22	137	6.23
10	Tianjin University of Traditional Chinese Medicine	China	22	322	14.64

### 3.5 Journal analysis

**Figure 5:**
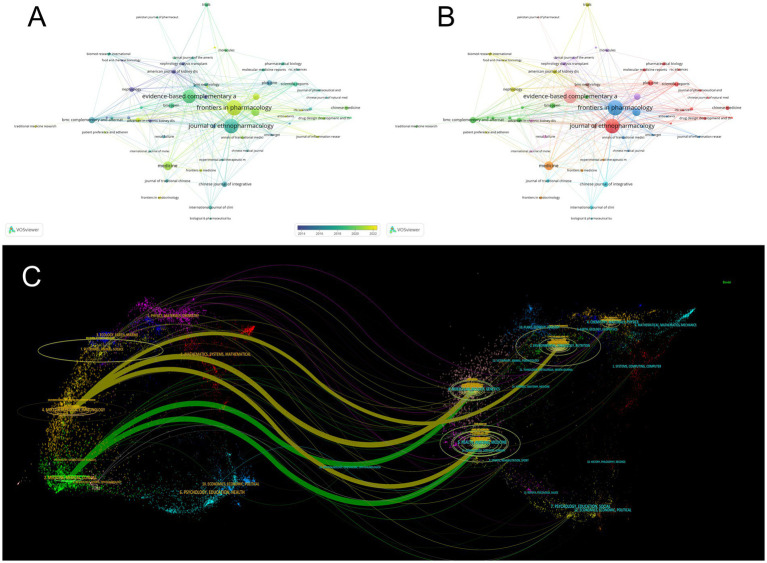
Analysis of journals. **(A)** Visual map showing influential journals in the study area, such as Frontiers in Pharmacology, Journal of Ethnopharmacology, and Evidence-Based Complementary. **(B)** Distribution graph indicating “Journal of Ethnopharmacology” has a long publication time, while “Frontiers in Pharmacology” publishes more recently. **(C)** Double-stacked graph revealing citation patterns between different fields of journals.

Among the articles included in this study, we drew a visual map of the participating journals (Figure 5A), where the size of the circle represents the number of published articles, from which it can be seen that Frontiers in Pharmacology, Journal of Ethnopharmacology, and Evidence-Based COMPLEMENTARY are the journals with high impact in this study area. All three journals are pharmacy related journals. Meanwhile, we presented the journals involved in the study in the form of a publication time distribution graph (Figure 5B), in which the color of the nodes changes from purple to yellow, representing the progression of time, which shows that “journal of ethnopharmacology,” as an influential journal in the field, has a long average publication time. In contrast, “Frontiers in Pharmacology have been involved in publishing more articles in recent years (Table 3). In addition, we analyzed the association between citing journals and cited journals using a journal double-stacked graph (Figure 5C). The diagram’s left side represents the citing journal, the right side denotes the cited journal, and the color-coded lines illustrate the connection between the citing and cited journals. The figure mainly shows that articles in molecular, biology, and immunology especially cited articles in molecular, biology and genetics, as well as articles in medicine and clinical medicine mainly cited articles related to life sciences and nursing in this collection. The focal points of these journals, such as pharmacology and ethnopharmacology, are closely related to the theme of this research, and their high influence in the relevant fields makes them the preferred journals for researchers to choose.

**Table 3:** Top 10 journals with the most publications.

Rank	Journal	Article counts	Percentage(426)	IF	Quartile in category
1	Frontiers in pharmacology	90	0.21	5.6	Q1
2	Journal of ethnopharmacology	81	0.19	5.4	Q1
3	Evidence-based complementary and alternative medicine	73	0.17	2.6	Q3
4	Medicine	35	0.08	1.6	Q3
5	Phytomedicine	30	0.07	7.9	Q1
6	Biomedicine & pharmacotherapy	21	0.05	7.5	Q1
7	BMC complementary and alternative medicine	19	0.04	2.4	Q3
8	Chinese journal of integrative medicine	15	0.04	2.9	Q3
9	Plos one	15	0.04	3.7	Q2
10	BMJ open	14	0.03	2.9	Q2

### 3.6 Author analysis

**Figure 6:**
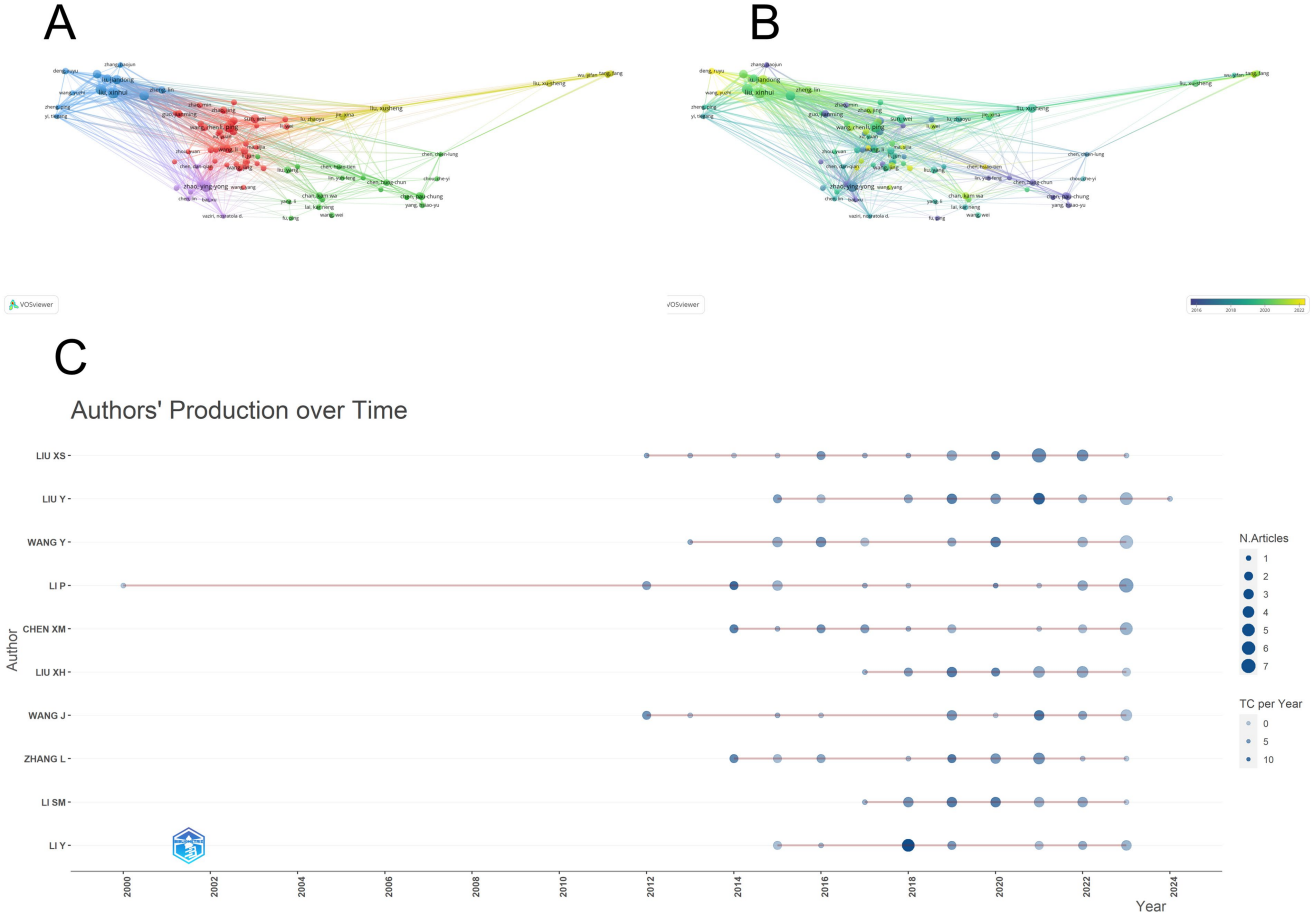
Analysis of co-authors. **(A)** Visual map showing author collaboration mainly in groups with limited cross-institutional cooperation. **(B)** Time map indicating high-impact Chinese scholars’ publication patterns before 2022. **(C)** Posting patterns of top authors, with most starting after 2012 and few contributing for over a decade.

Through data calculation, a total of 5,358 authors participated in the submission; Figure 6A lists the co-authorship network of authors who published more than 4 papers, and plots the co-authorship network with 74 authors participating. The connecting line indicates the cooperation between individual authors, the size of the circle shows the number of published papers, and the color of the node changes from purple to yellow to indicate the time progression. It can be seen that the collaboration between authors is mainly in the form of groups, and these authors mostly use institutions as a mode of collaborative research, with relatively little cooperation across institutions. Figure 6B shows the change in authors’ publication time, which shows that several Chinese scholars with high impact published more articles before 2022, and these scholars were involved in publishing articles in all periods. Table 4 presents the 10 authors deemed most pertinent within the realm of Chinese medicine research, all of whom have authored nine or more articles. Collectively, these 10 authors have contributed 131 publications, representing 11.36% of the total literature in this field. Notably, all authors hail from China and have made substantial impacts on the field. Leading the cohort is Liu xinhui, with 17 published papers, followed closely by other esteemed scholars. The distribution of publication years among the top 10 most relevant authors is depicted in Figure 6C. The majority of these authors commenced publishing after 2012, with only a small number having made contributions to the field for over a decade. The co-authorship network in this field shows a certain degree of group collaboration, and Chinese scholars play a leading role. However, there is still room for further strengthening international collaboration and promoting more diverse and extensive research cooperation.

**Table 4:** Top 10 authors with the most publications.

Rank	Author	Count	Location	Rank	Co-cited author	Citation
1	Liu xinhui	17	China	1	Zhao, YY	350
2	Li ping	16	China	2	Levey, AS	147
3	Li shunmin	16	China	3	zhang, L	105
4	Zhao yingyong	16	China	4	Zhong, YF	103
5	Chen jianping	13	China	5	Chen, DQ	102
6	Lu jiandong	12	China	6	Wang, M	91
7	Liu xusheng	11	China	7	Jha, V	84
8	Sun wei	11	China	8	Zhang, ZH	82
9	Huang shiying	10	China	9	Chen, H	74
10	Chen xiangmei	9	China	10	Wang, Y	74

### 3.7 Visual analysis of keyword co-occurrence

**Figure 7:**
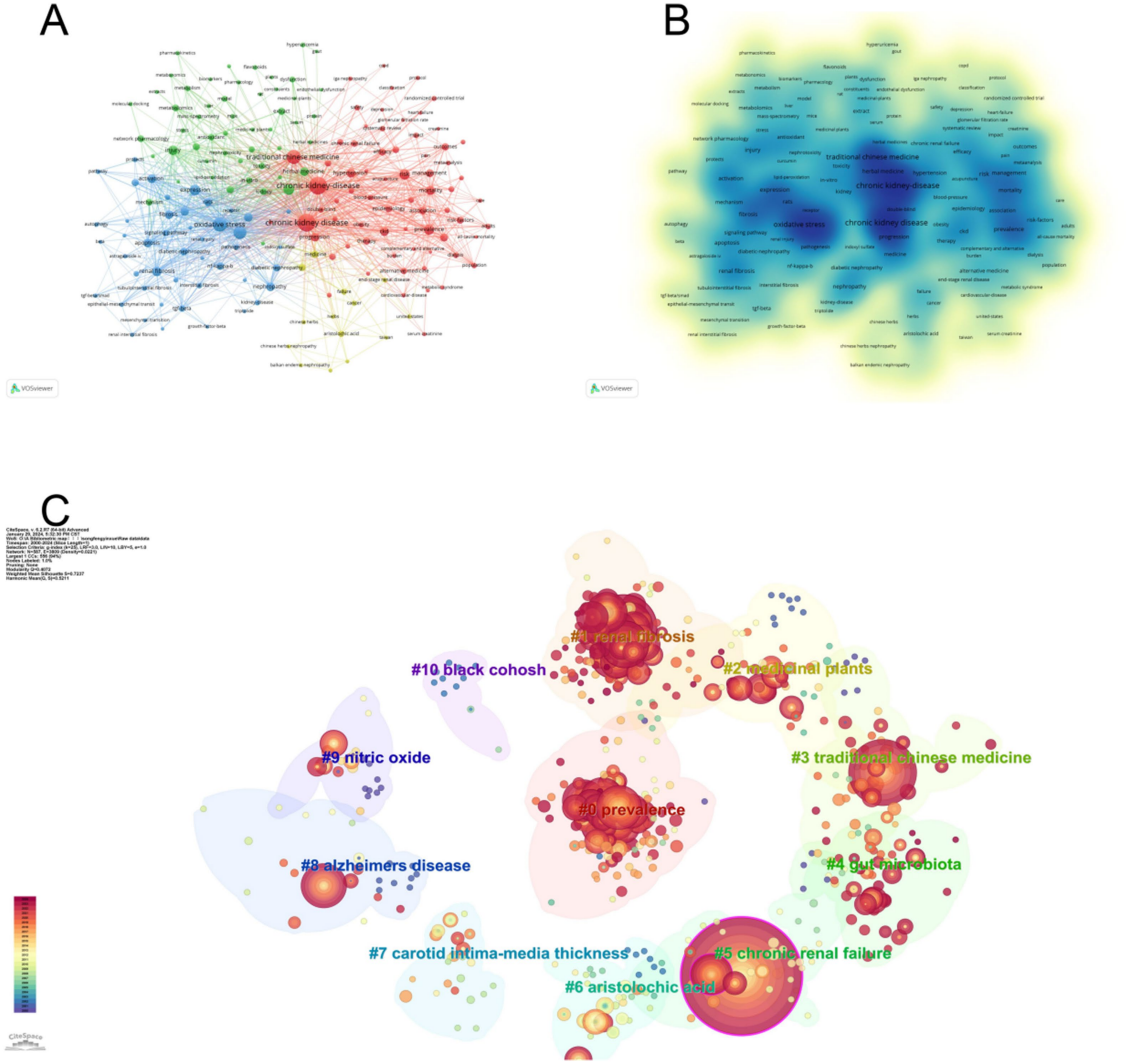
Keyword visualization analysis. **(A)** Visualization map of keywords, showing that “chronic kidney disease,” “oxidative stress,” “traditional chinese medicine,” and “inflammation” are frequently used. **(B)** Keyword density map, indicating the frequency of keyword occurrence. **(C)** Keyword clustering map, revealing 11 clusters, with #0prevalence containing the most keywords and associations.

**Figure 8:**
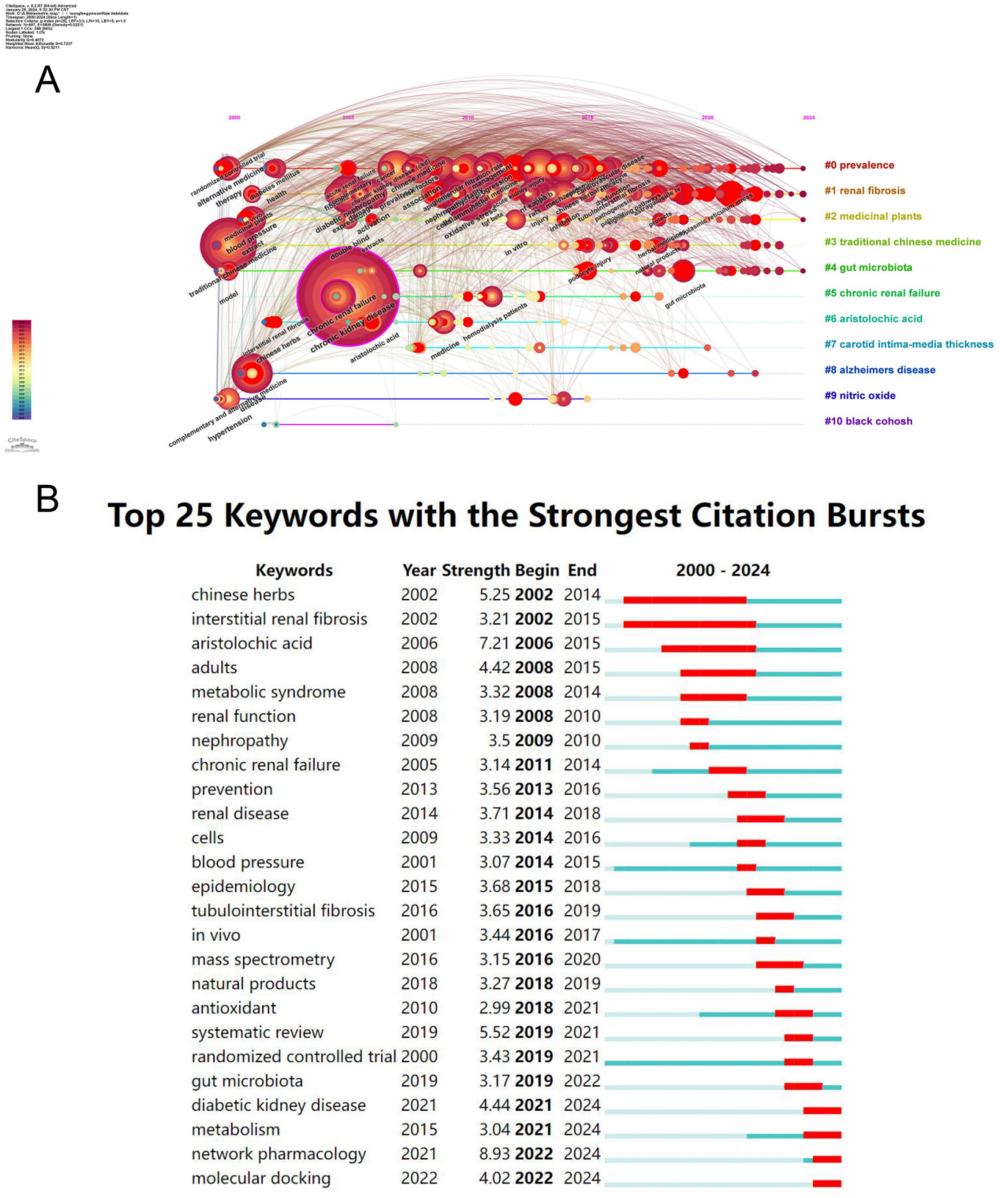
Keyword Analysis. **(A)** Timeline graph showing keyword distribution and evolution, with #0prevalence more evenly distributed and some keywords used consistently. **(B)** Outbreak graph indicating “network pharmacology” has high burst intensity in 2022–2024, and “interstitial renal fibrosis” has the longest burst duration.

The keywords of an article can represent the topic and main ideas of the article, and by constructing a recurrence map of keywords, the research hotspots and trends in the field can be described. We used VOSviewer to visualize and analyze the keywords that appeared 10 times or more in the articles, and obtained a total of 180 keywords (Figures 7A,B). The larger or darker color of the circle represented by a keyword indicates that the keyword occurs more frequently and defines a research hotspot in the field; moreover, the connecting line between two keywords indicates that they co-occur in the same article, and its thickness is proportional to the frequency of co-occurrence. It can be seen that the most frequently used keywords in this research field are “chronic kidney disease,” “oxidative stress,” “traditional Chinese medicine,” and “inflammation,” and the keywords with more than 20 occurrences are roughly divided into four color groups (Table 5). The keyword “chronic kidney disease” is at the center of all articles. The keyword “chronic kidney disease” was at the center of all the articles. We performed cluster analysis of the keywords by Citespace software, and 11 clusters were calculated, which were #0prevalence, #1renal fibrosis, #2medicinal plants, #3 traditional Chinese medicine, #4gut microbiota, #5chronic renal failure, #6aristolochic acid, #7carotid intima-media thickness, #8alzheimers disease, #9nitric oxide, #10 black cohosh (Figure 7C), where the keyword cluster #0prevalence contains the highest number of keywords and the highest number of associations with other keywords, i.e., the highest number of simultaneous occurrences in a document. In order to visualize the change of keywords over time, we constructed a keyword timeline graph (Figure 8A); the size of the k node represents the frequency of keyword occurrence, the color of the circle depicts the average time of occurrence, and the color changes from purple to red as the chronological order changes from 2000 to 2024. It can be seen in the graph that the distribution of keywords in cluster #0prevalence is more even, and the distribution of keywords in the other the keyword distribution of cluster #0prevalence is more dispersed, and some keywords used before 2005 are still consistently used over a long period. It can be seen that cluster #0prevalence has the richest keyword evolution and the most resounding impact on the clusters. In addition, we calculated the bursting trend of keyword occurrences by Citespace software. Figure 8B shows the 25 keywords with the highest bursting intensity, with the blue line representing the length of occurrences and the red line representing the length of keyword bursts. Among these 25 keywords, the keyword with the highest burst intensity is “network pharmacology,” which mainly appears between 2022 and 2024, and belongs to the explosive research hotspot; the keyword with the most prolonged burst duration is “interstitial renal fibrosis,” which indicates that this research direction is a hot research direction for scholars for a long time. The most prolonged duration of the outbreak is “interstitial renal fibrosis,” indicating that this research direction has been a hot research direction for scholars for an extended period, but the research intensity gradually decreased after 2010. The analysis of keywords and clusters reveals the key research topics and trends in the field of chronic kidney disease and traditional Chinese medicine. The centrality of “chronic kidney disease” and the distribution of other keywords provide an overview of the research landscape. The identification of emerging hotspots like “network pharmacology” and sustained research directions like “interstitial renal fibrosis” is crucial for guiding future research.

**Table 5:** Top 20 high-frequency keywords.

Rank	Keyword	Counts	Rank	Keyword	Counts
1	Chronic kidney-disease	224	11	Injury	62
2	Chronic kidney disease	217	12	Mortality	59
3	Oxidative stress	146	13	Fibrosis	56
4	Traditional Chinese medicine	141	14	Apoptosis	55
5	Inflammation	119	15	CKD	55
6	Disease	82	16	Mechanisms	55
7	Expression	78	17	Activation	54
8	Prevalence	76	18	Nephropathy	54
9	Risk	73	19	Herbal medicine	53
10	Renal fibrosis	65	20	Hypertension	52

## 4 Discussion

TCM offers effective treatment for chronic diseases by using holistic concepts, personalized treatment, various methods, and extensive clinical experience. To enhance TCM’s effectiveness in treating CKD, its application in CKD needs improvement (17). In the past 24 years, research on TCM intervention for CKD has significantly increased, with a sharp upward trend since 2019 and the most articles published in 2022. This indicates that it has become a hot topic in the scientific research field, likely due to China’s economic growth and increased attention to TCM. The implementation of the “Law of the People’s Republic of China on Traditional Chinese Medicine” in 2017 enhanced the legal status of TCM in China and its global influence, attracting more international researchers and promoting its research and application globally (29, 30).

China’s leading position in research on the treatment of CKD with TCM is well-documented and corroborated by a plethora of scholarly articles. The preponderance of literature emanating from China, coupled with the high citation rates it garners, underscores the country’s significant contributions to this field (22). Our study further revealed growth trends in other countries (e.g., the United States, India, Iran, Japan and Korea). The increased research output in the United States on the application of TCM for CKD treatments mirrors a national commitment to integrative medical approaches, underscored by regulatory endorsements from the FDA for herbal medicine trials (31), the establishment of TCM legislative frameworks (32), and an escalating market demand for TCM therapeutics (33). The top 10 countries in terms of publication volume include not only Asian countries such as India, Iran, Japan, and South Korea but also European countries such as the United Kingdom, Italy, Canada, and Australia. Indian traditional medicine such as Ayurvedic medicine has herbal therapies, yoga, etc., which are also widely used in TCM (34). The two have learned from, borrowed from and developed each other in medical theories and drugs (35). Our research has identified a notable upsurge in TCM research in India and Iran, a trend that is intrinsically linked to the escalating domestic demand for herbal medicines and the supportive governmental funding initiatives. The persistent growth trajectory of these nations within our study underscores that international collaboration, policy endorsement, and scholarly engagement are the cornerstones that have elevated them as leading figures in the realm of TCM for CKD treatment. This indicates that the research on TCM intervention in CKD is receiving increasing international attention. This global trend will further promote the development of TCM and promote the recognition and application of TCM worldwide.

The analysis shows that five of the top 10 most prolific institutions are TCM universities, which are predominantly located in five economically robust regions: Guangzhou, Beijing, Shanghai, Nanjing, and Tianjin. This suggests a link between economic vitality and the flourishing of TCM research. The China Academy of Chinese Medicine stands out for its significant contributions, reflecting its status as a leading research institution in the field. At the same time, comprehensive universities, such as Peking University, Fudan University, China Medical University, and Capital Medical University, among the top contributors indicate a growing interest in TCM from institutions traditionally associated with Western medicine. This interdisciplinary collaboration could pave the way for novel treatments by combining traditional insights with modern scientific approaches.

By conducting an in-depth analysis of the top 10 journals with the most publications, we can gain a deeper understanding of the current development trends and dynamics in the field of academic publishing. Our findings reveal that “Frontiers in Pharmacology,” “Journal of Ethnopharmacology,” and “Evidence-Based Complementary and Alternative Medicine” are the most prolific journals in publishing research on TCM and CKD. These observations align with those of Xu et al. (22), who noted that these periodicals are particularly receptive to studies on traditional Chinese medicine and integrative healthcare, boasting significant visibility and global impact. Notably, “Frontiers in Pharmacology” has emerged as an ideal platform for publishing TCM research, owing to its recent focus on emerging fields such as network pharmacology. Furthermore, the thematic alignment of “Journal of Ethnopharmacology” with traditional medicine research has solidified its prominent role in showcasing scientific achievements within the TCM domain. However, it is worth noting that among the numerous academic journals in China, only the Chinese Journal of Integrated Traditional Chinese and Western Medicine stands out among the top 10 in terms of publication volume. Despite the high volume of academic research in China, Chinese academic journals have low influence and attention internationally. To improve this, Chinese journals need to enhance their quality and increase international collaborations for greater global recognition.

TCM has a significant historical background and extensive expertise in the treatment of CKD (36), but its mechanism of action is often overlooked or misunderstood. Through in-depth analysis and research on the articles of the most published authors, we found that researchers are more inclined to study the mechanism of a single drug or TCM compound in the treatment of CKD. Only when the world can understand and accept TCM, can the great potential of TCM in the treatment of various diseases, including CKD, be genuinely realized. Professor Liu xinhui’s research primarily focuses on the Jian-Pi-Yi-Shen Formula (JPYSF), which effectively improves renal function and structure in CKD rats through various mechanisms such as modulating the mitochondrial quality control network, tryptophan metabolism, aryl hydrocarbon receptor signaling, renal glucose metabolism pathways, and by activating SIRT3 and enhancing antioxidant effects (37–40). Additionally, Professor Liu has explored the relationship between the gut microbiome’s response to JPYSF and the resulting renal improvements (41), as well as the therapeutic mechanisms of various traditional Chinese herbs like astragalus, Danshen, and naringin in treating CKD (42–44). Researcher Li Ping is more focused on the clinical efficacy research of TCM in treating CKD, such as multi-center randomized controlled clinical trials on the efficacy and safety of TCM compound preparations in treating primary glomerulonephritis (45), the clinical efficacy of Shen Hua Tablets against mesangial cell proliferation and nephritis (46), and a clinical controlled study comparing TCM with losartan potassium in treating IgA nephropathy (47). He also pays attention to the underlying mechanisms of TCM in treating CKD, such as exploring the molecular mechanisms of TCM compounds in treating diabetic nephropathy based on network pharmacology (48), and investigating the therapeutic mechanisms of TCM in gouty nephropathy via the NF-κB signaling pathway (49). This aligns with the research of Denise Mafra (50), who also observed the growing interest in the role of the gut microbiome in chronic diseases. However, our study also found that ‘network pharmacology’, as an emerging methodology, is gradually becoming a research hotspot in this field, which has not been fully reflected in other studies. Renal fibrosis is a frequent occurrence in the advancement of CKD to ESRD, and it is a significant histopathological indication of CKD (51). Renal fibrosis is a series of changes, including excessive accumulation of extracellular matrix, tubular epithelial-mesenchymal transition (EMT), activation of fibroblasts, infiltration of immune cells, and apoptosis of renal cells, ultimately leading to renal dysfunction and even renal failure (52, 53). However, there is no effective treatment for renal fibrosis at present (54). Dihydroartemisinin (DHA) exhibits renoprotective effects and reverses renal fibrosis by targeting DNA methyltransferase 1 (DNMT1) to reverse Klotho repression, inhibiting the Wnt/*β*-catenin and TGF-β/Smad signaling pathways, and providing evidence for its potential clinical application in treating renal fibrosis (55). Tongluo Yishen Decoction can reduce oxidative stress and regulate mitochondrial autophagy, improving mitochondrial dynamics, thereby reducing renal injury, protecting renal function, and reducing renal fibrosis (56). Shenkang injection exhibits anti-fibrotic and renal protective effects in *in vivo* and *in vitro* animal models and cell experiments by inhibiting the TGF-β/Smad3 signaling pathway, regulating mitochondrial autophagy, reducing oxidative stress and inflammatory responses, providing potential molecular mechanisms and clinical application prospects for the treatment of CKD (57–59). In addition, the relationship between gut microbiota and CKD has also become one of the research hotspots. Studies have shown that the imbalance of the gut microbiota disrupts the integrity of the intestinal barrier, causing bacterial displacement and metabolite accumulation, which leads to abnormal activation of immune cells and inflammatory response, and these factors can lead to renal parenchymal injury (60). Therefore, a deep study of the relationship between the intestinal microbiome and CKD is of great significance for the prevention and treatment of CKD. It was found that the interaction with the intestinal bacteria of normal and chronic renal failure rats can regulate the intestinal bacterial composition through metabolic transformation (61). Dahuang Gancao Decoction can reduce the accumulation of uremic toxin in animal models of renal failure in the regulation of intestinal flora (62). Yiqihuoxuejiangzhuo Formula protects the heart function of CKD mouse models by regulating intestinal flora and inhibiting the activation of NLRP3 inflammasome, demonstrating its potential mechanism of heart and kidney protection (63). The relationship between nitric oxide and CKD has also received widespread attention. Nitric oxide (NO) plays a complex and critical role in the occurrence and development of CKD. In CKD, the production and activity of NO in the kidney may be affected, which may lead to renal vascular dysfunction and worsening of renal injury (64, 65). The level of NO anabolic compounds can be used as an essential biomarker to predict cardiovascular outcomes in patients with CKD (66, 67). Increasing the production and activity of NO may help improve renal function and prognosis in patients with CKD (68). Multiple Chinese herbal ingredients have a positive effect on the production and activity of NO, which may be a new target for the treatment of CKD with TCM in the future (69–72).

Our study is subject to several limitations. Firstly, the focus on the Web of Science database may have resulted in the omission of pertinent literature not indexed within this platform, including non-English publications and those appearing in less prominent journals. Secondly, the reliance on publication and citation counts as metrics for assessing research impact fails to encapsulate the qualitative dimensions of scholarly work, such as the intricacy of the research or its practical applications. Thirdly, the temporal scope of our analysis, which covers the period from 2000 to 2024, may not adequately represent the most recent advancements or offer a thorough historical perspective.

## 5 Conclusion

The remarkable growth in TCM research on CKD signals a transition towards integrative medicine, reflecting the international medical community’s interest in TCM’s holistic treatment approaches. As TCM and Western medical institutions collaborate, there is potential for a more diverse therapeutic panorama for CKD patients. We suggest stronger international partnerships to validate TCM’s efficacy through clinical trials and incorporate it into CKD treatment guidelines. Future studies should delve into TCM’s molecular mechanisms, particularly in network pharmacology and gut microbiota, to innovate CKD treatments. Policymakers should also consider the economic aspects of CKD management and promote sustainable access to TCM. Our study not only analyzes the current TCM research landscape but also emphasizes the need for continuous research and innovation to enhance TCM’s role in combating CKD globally.

## Data Availability

The original contributions presented in the study are included in the article/supplementary material, further inquiries can be directed to the corresponding author.

## References

[1] Gu X Yang H Sheng X Ko YA Qiu C Park J . Kidney disease genetic risk variants alter lysosomal beta-mannosidase (MANBA) expression and disease severity. Sci Transl Med. (2021) 13:eaaz1458. doi: 10.1126/scitranslmed.aaz1458, PMID: 33441424 PMC8627675

[2] Wei J Zhang J Wang L Cha BJ Jiang S Liu R. A new low-nephron CKD model with hypertension, progressive decline of renal function, and enhanced inflammation in C57BL/6 mice. Am J Physiol Ren Physiol. (2018) 314:F1008–19. doi: 10.1152/ajprenal.00574.2017, PMID: 29412703 PMC6031904

[3] Kim DH Park JS Choi HI Kim CS Bae EH Ma SK . The critical role of FXR is associated with the regulation of autophagy and apoptosis in the progression of AKI to CKD. Cell Death Dis. (2021) 12:320. doi: 10.1038/s41419-021-03620-z, PMID: 33767132 PMC7994637

[4] Aranda-Rivera AK Cruz-Gregorio A Pedraza-Chaverri J Scholze A. Nrf2 activation in chronic kidney disease: promises and pitfalls. Antioxidants (Basel, Switzerland). (2022) 11:1112. doi: 10.3390/antiox11061112, PMID: 35740009 PMC9220138

[5] Koide T Mandai S Kitaoka R Matsuki H Chiga M Yamamoto K . Circulating extracellular vesicle-propagated microRNA signature as a vascular calcification factor in chronic kidney disease. Circ Res. (2023) 132:415–31. doi: 10.1161/CIRCRESAHA.122.321939, PMID: 36700539

[6] Zhao H Ma SX Shang YQ Zhang HQ Su W. microRNAs in chronic kidney disease. Clin Chim Acta. (2019) 491:59–65. doi: 10.1016/j.cca.2019.01.008, PMID: 30639583

[7] Ruiz-Ortega M Rayego-Mateos S Lamas S Ortiz A Rodrigues-Diez RR. Targeting the progression of chronic kidney disease. Nat Rev Nephrol. (2020) 16:269–88. doi: 10.1038/s41581-019-0248-y 32060481

[8] Bikbov B Purcell CA Levey AS. Global, regional, and national burden of chronic kidney disease, 1990-2017: a systematic analysis for the global burden of disease study 2017. Lancet. (2020) 395:709–33. doi: 10.1016/S0140-6736(20)30045-3, PMID: 32061315 PMC7049905

[9] Chen TK Knicely DH Grams ME. Chronic kidney disease diagnosis and management: a review. JAMA. (2019) 322:1294–304. doi: 10.1001/jama.2019.14745, PMID: 31573641 PMC7015670

[10] van Oosten M Logtenberg S Leegte M Bilo H Mohnen SM Hakkaart-van Roijen L . Age-related difference in health care use and costs of patients with chronic kidney disease and matched controls: analysis of Dutch health care claims data. Nephrol Dial Transplant. (2020) 35:2138–46. doi: 10.1093/ndt/gfz146, PMID: 31598728 PMC7716809

[11] Elshahat S Cockwell P Maxwell AP Griffin M O'Brien T O'Neill C. The impact of chronic kidney disease on developed countries from a health economics perspective: a systematic scoping review. PLoS One. (2020) 15:e0230512. doi: 10.1371/journal.pone.0230512, PMID: 32208435 PMC7092970

[12] Kim KH Lee MS Kim TH Kang JW Choi TY Lee JD. Acupuncture and related interventions for symptoms of chronic kidney disease. Cochrane Database Syst Rev. (2016) 2016:CD009440. doi: 10.1002/14651858.CD009440.pub2, PMID: 27349639 PMC8406453

[13] Lin LL Wang YH Lai CY Chau CL Su GC Yang CY . Systems biology of meridians, acupoints, and chinese herbs in disease. Evid Based Complement Alternat Med. (2012) 2012:372670. doi: 10.1155/2012/372670, PMID: 23118787 PMC3483864

[14] Xiong W He FF You RY Xiong J Wang YM Zhang C . Acupuncture application in chronic kidney disease and its potential mechanisms. Am J Chin Med. (2018) 46:1169–85. doi: 10.1142/S0192415X18500611, PMID: 30286626

[15] Wang Y Feng Y Li M Yang M Shi G Xuan Z . Traditional Chinese medicine in the treatment of chronic kidney diseases: theories, applications, and mechanisms. Front Pharmacol. (2022) 13:917975. doi: 10.3389/fphar.2022.917975, PMID: 35924053 PMC9340222

[16] Isnard Bagnis C Deray G Baumelou A Le Quintrec M Vanherweghem JL. Herbs and the kidney. Am J Kidney Dis. (2004) 44:1–11. doi: 10.1053/j.ajkd.2004.02.009, PMID: 15211432

[17] Wojcikowski K Johnson DW Gobé G. Medicinal herbal extracts -- renal friend or foe? Part one: the toxicities of medicinal herbs. Nephrology (Carlton). (2004) 9:313–8. doi: 10.1111/j.1440-1797.2004.00310.x 15504145

[18] Donthu N Kumar S Mukherjee D Pandey N Lim WM. How to conduct a bibliometric analysis: An overview and guidelines. J Bus Res. (2021) 133:285–96. doi: 10.1016/j.jbusres.2021.04.070

[19] Ai S Li Y Zheng H Wang Z Liu W Tao J . Global research trends and hot spots on autophagy and kidney diseases: a bibliometric analysis from 2000 to 2022. Front Pharmacol. (2023) 14:1275792. doi: 10.3389/fphar.2023.1275792, PMID: 38099142 PMC10719858

[20] Sun HL Bai W Li XH Huang H Cui XL Cheung T . Schizophrenia and inflammation research: a bibliometric analysis. Front Immunol. (2022) 13:907851. doi: 10.3389/fimmu.2022.907851, PMID: 35757702 PMC9219580

[21] Zhang F Liu S Bai Y Huang L Zhong Y Li Y. Exercise training and chronic kidney disease: characterization and bibliometrics of citation classics of clinical intervention trials. Ren Fail. (2024) 46:2349187. doi: 10.1080/0886022X.2024.2349187, PMID: 38721893 PMC11085942

[22] Xu Y Chen J Wang H Lu Y. Research and application of herbal medicine in the treatment of chronic kidney disease since the 21st century: a visualized bibliometric analysis. Front Pharmacol. (2022) 13:971113. doi: 10.3389/fphar.2022.971113, PMID: 36249821 PMC9561987

[23] Gholampour B Gholampour S Noruzi A. Research trend analysis of information science in France based on total, cited and uncited publications: a scientometric and altmetric analysis. Inform. (2022) 1:7–26. doi: 10.3389/fmed.2022.898624

[24] Wan Y Shen J Ouyang J Dong P Hong Y Liang L . Bibliometric and visual analysis of neutrophil extracellular traps from 2004 to 2022. Front Immunol. (2022) 13:1025861. doi: 10.3389/fimmu.2022.1025861, PMID: 36341351 PMC9634160

[25] Chen YM Wang XQ. Bibliometric analysis of exercise and neuropathic pain research. J Pain Res. (2020) 13:1533–45. doi: 10.2147/JPR.S258696, PMID: 32612381 PMC7323814

[26] Li C Jin Y Homapour E. A scientometric review of hotspots and emerging trends in sustainable business model. Heliyon. (2023) 9:e18446. doi: 10.1016/j.heliyon.2023.e18446, PMID: 37560644 PMC10407053

[27] Gholampour B Noruzi A Elahi A Barranco Gil D Gholampour S. Who are the key figures in grand Tours cycling events publications? A systematic review of main themes. Global Knowledge, Memory and Commun. (2024) 1:7–26. doi: 10.1108/GKMC-12-2023-0472

[28] Gholampour S Gholampour B Elahi A Noruzi A Saboury AA Hassan S . From mega-events hosting to scientific leadership: a seven-decade scientometric analysis of pioneer countries. Cogent Soc Sci. (2023) 9:2210398. doi: 10.1080/23311886.2023.2210398

[29] Sang BS Liu XT. Review on construction of legal system of traditional Chinese medicine in China. Zhonghua Yi Shi Za Zhi. (2017) 47:3–13. doi: 10.3760/cma.j.issn.0255-7053.2017.01.001, PMID: 28316201

[30] Yuan H Ma Q Cui H Liu G Zhao X Li W . How can synergism of traditional medicines benefit from network pharmacology. Molecules. (2017) 22:1135. doi: 10.3390/molecules22071135, PMID: 28686181 PMC6152294

[31] Lu WI Lu DP. Impact of Chinese herbal medicine on American society and health care system: perspective and concern. Evidence-Based Complementray Alternative Med. (2014) 547–606:251891. doi: 10.1155/2014/251891, PMID: 24719641 PMC3955605

[32] Fan AY Stumpf SH Faggert Alemi S Matecki A. Distribution of licensed acupuncturists and educational institutions in the United States at the start of 2018. Complement Ther Med. (2018) 41:295–301. doi: 10.1016/j.ctim.2018.10.015, PMID: 30477857

[33] Jin LL Zheng J Honarvar NM Chen X. Traditional Chinese medicine in the United States: current state, regulations, challenges, and the way forward. Traditional Med Modern Med. (2021) 3:77–84. doi: 10.1142/S2575900020100023, PMID: 38200116

[34] Li X Wu L Wu R Sun M Fu K Kuang T . Comparison of medicinal preparations of Ayurveda in India and five traditional medicines in China. J Ethnopharmacol. (2022) 284:114775. doi: 10.1016/j.jep.2021.114775, PMID: 34742863

[35] Wu L Chen W Wang Z. Traditional Indian medicine in China: the status quo of recognition, development and research. J Ethnopharmacol. (2021) 279:114317. doi: 10.1016/j.jep.2021.114317, PMID: 34111541

[36] Wojcikowski K Johnson DW Gobe G. Herbs or natural substances as complementary therapies for chronic kidney disease: ideas for future studies. J Lab Clin Med. (2006) 147:160–6. doi: 10.1016/j.lab.2005.11.011, PMID: 16581343

[37] Liu XH Chen JP Liu XY Wang DT Zheng P Qi AR . Jian-pi-Yi-Shen formula ameliorates chronic kidney disease: involvement of mitochondrial quality control network. BMC Complement Altern Med. (2018) 18:340. doi: 10.1186/s12906-018-2395-2, PMID: 30572886 PMC6302435

[38] Liu XH Deng RY Chen YL Huang SY Lu JD Zheng L . Jian-pi-Yi-Shen formula improves adenine-induced chronic kidney disease via regulating tryptophan metabolism and aryl hydrocarbon receptor signaling. Front Pharmacol. (2022) 13:13. doi: 10.3389/fphar.2022.922707, PMID: 35865941 PMC9294467

[39] Liu XH Deng RY Wei X Wang YZ Weng JL Lao YL . Jian-pi-Yi-Shen formula enhances perindopril inhibition of chronic kidney disease progression by activation of SIRT3, modulation of mitochondrial dynamics, and antioxidant effects. Biosci Rep. (2021) 41:41. doi: 10.1042/BSR20211598, PMID: 34633033 PMC8536834

[40] Liu XH Gao LW Huang X Deng RY Wei X Lu JD . Lipidomics reveals the potential mechanism of honokiol against adenine-induced chronic kidney disease. Front Pharmacol. (2022) 13:13. doi: 10.3389/fphar.2022.1019629, PMID: 36313325 PMC9614281

[41] Zheng L Chen S Wang FC Huang SY Liu XH Yang XL . Distinct responses of gut microbiota to Jian-pi-Yi-Shen decoction are associated with improved clinical outcomes in 5/6 Nephrectomized rats. Front Pharmacol. (2020) 11:11. doi: 10.3389/fphar.2020.00604, PMID: 32435197 PMC7219274

[42] Huang X Gao LW Deng RY Peng Y Wu SS Lu JD . Huangqi-Danshen decoction reshapes renal glucose metabolism profiles that delays chronic kidney disease progression. Biomed Pharmacother. (2023) 164:114989. doi: 10.1016/j.biopha.2023.114989, PMID: 37315436

[43] Liu XH Zhang B Huang SY Wang FC Zheng L Lu JD . Metabolomics analysis reveals the protection mechanism of Huangqi-Danshen decoction on adenine-induced chronic kidney disease in rats. Front Pharmacol. (2019) 10:10. doi: 10.3389/fphar.2019.00992, PMID: 31551789 PMC6747014

[44] Zhang ZY Fang JA Sun DL Zheng YQ Liu XH Li H . Study on the mechanism of Radix Astragali against renal aging based on network pharmacology. Evid Based Complement Alternat Med. (2022) 2022:1–13. doi: 10.1155/2022/6987677, PMID: 36561604 PMC9767736

[45] Zhang L Li P Xing CY Zhao JY He YN Wang JQ . Efficacy and safety of Abelmoschus manihot for primary glomerular disease: a prospective, multicenter randomized controlled clinical trial. Am J Kidney Dis. (2014) 64:57–65. doi: 10.1053/j.ajkd.2014.01.431, PMID: 24631042

[46] He JY Peng F Chang JK Zhao YH Qu YL Liu JA . The therapeutic effect of Shenhua tablet against mesangial cell proliferation and renal function in mesangial proliferative glomerulonephritis. Biomed Pharmacother. (2023) 165:F95–F102. doi: 10.1016/j.biopha.2023.115233, PMID: 37536037

[47] Li P Chen YZ Lin HL Ni ZH Zhan YL Wang R . *Abelmoschus manihot* - a traditional Chinese medicine versus losartan potassium for treating IgA nephropathy: study protocol for a randomized controlled trial. Trials. (2017) 18:18. doi: 10.1186/s13063-016-1774-6, PMID: 28395659 PMC5387231

[48] Chen K Deng YY Shang SL Li P Liu LC Chen XM. Network pharmacology-based investigation of the molecular mechanisms of the Chinese herbal formula Shenyi in the treatment of diabetic nephropathy. Front Med. (2022) 9:9. doi: 10.3389/fmed.2022.898624, PMID: 35755045 PMC9226379

[49] Liu P Ma GJ Wang Y Wang LF Li P. Therapeutic effects of traditional Chinese medicine on gouty nephropathy: based on NF-ΚB signalingpathways. Biomed Pharmacother. (2023) 158:158. doi: 10.1016/j.biopha.2022.114199, PMID: 36916428

[50] Josef B Archie B Ashok C Gerald P Sapra KJ Roger C . Advanced chronic kidney disease in non-valvular atrial fibrillation: extending the utility of R2CHADS2 to patients with advanced renal failure. Clin Kidney J. (2015) 8:226–31. doi: 10.1093/ckj/sfv006, PMID: 25815182 PMC4370306

[51] Liang S Wu YS Li DY Tang JX Liu HF. Autophagy and renal fibrosis. Autophagy and Renal Fibrosis Aging Dis. (2022) 13:712–31. doi: 10.14336/AD.2021.1027, PMID: 35656109 PMC9116923

[52] Humphreys BD. Mechanisms of renal fibrosis. Annu Rev Physiol. (2018) 80:309–26. doi: 10.1146/annurev-physiol-022516-034227, PMID: 29068765

[53] Nastase MV Zeng-Brouwers J Wygrecka M Schaefer L. Targeting renal fibrosis: mechanisms and drug delivery systems. Adv Drug Deliv Rev. (2018) 129:295–307. doi: 10.1016/j.addr.2017.12.019, PMID: 29288033

[54] Chen YY Chen XG Zhang S. Druggability of lipid metabolism modulation against renal fibrosis. Acta Pharmacol Sin. (2022) 43:505–19. doi: 10.1038/s41401-021-00660-1, PMID: 33990764 PMC8888625

[55] Zhou W Chen MM Liu HL Si ZL Wu WH Jiang H . Dihydroartemisinin suppresses renal fibrosis in mice by inhibiting DNA-methyltransferase 1 and increasing klotho. Acta Pharmacol Sin. (2022) 43:2609–23. doi: 10.1038/s41401-022-00898-3, PMID: 35347248 PMC9525601

[56] Jia Q Han L Zhang X Yang W Gao Y Shen Y . Tongluo Yishen decoction ameliorates renal fibrosis via regulating mitochondrial dysfunction induced by oxidative stress in unilateral ureteral obstruction rats. Front Pharmacol. (2021) 12:762756. doi: 10.3389/fphar.2021.762756, PMID: 34712143 PMC8545824

[57] Hao J Huang X Guan J Feng J Li D Cao S . Shenkang injection protects against renal fibrosis by reducing perforin expression through the STING/TBK1/IRF3 signaling pathways in natural killer cells. Phytomedicine. (2022) 104:154206. doi: 10.1016/j.phymed.2022.154206, PMID: 35724525

[58] Luo LP Suo P Ren LL Liu HJ Zhang Y Zhao YY. Shenkang injection and its three Anthraquinones ameliorates renal fibrosis by simultaneous targeting IƙB/NF-ƙB and Keap1/Nrf2 signaling pathways. Front Pharmacol. (2021) 12:800522. doi: 10.3389/fphar.2021.800522, PMID: 35002735 PMC8729217

[59] Wu X Guan Y Yan J Liu M Yin Y Duan J . ShenKang injection suppresses kidney fibrosis and oxidative stress via transforming growth factor-β/Smad3 signalling pathway in vivo and in vitro. J Pharm Pharmacol. (2015) 67:1054–65. doi: 10.1111/jphp.12412, PMID: 25864844

[60] Chi M Ma K Wang J Ding Z Li Y Zhu S . The immunomodulatory effect of the gut microbiota in kidney disease. J Immunol Res. (2021) 2021:5516035–16. doi: 10.1155/2021/5516035, PMID: 34095319 PMC8140847

[61] Cai H Su S Li Y Zhu Z Guo J Zhu Y . Danshen can interact with intestinal bacteria from normal and chronic renal failure rats. Biomed Pharmacother. (2019) 109:1758–71. doi: 10.1016/j.biopha.2018.11.047, PMID: 30551430

[62] Han WB Liu YL Wan YG Sun W Tu Y Yang JJ . Pathomechanism and treatment of gut microbiota dysbiosis in chronic kidney disease and interventional effects of Chinese herbal medicine. Zhongguo Zhong Yao Za Zhi. (2017) 42:2425–32. doi: 10.19540/j.cnki.cjcmm.20170609.014, PMID: 28840678

[63] Liu T Lu X Gao W Zhai Y Li H Li S . Cardioprotection effect of Yiqi-Huoxue-Jiangzhuo formula in a chronic kidney disease mouse model associated with gut microbiota modulation and NLRP3 inflammasome inhibition. Biomed Pharmacother. (2022) 152:113159. doi: 10.1016/j.biopha.2022.113159 35661533

[64] Baylis C. Nitric oxide deficiency in chronic kidney disease. Am J Physiol Ren Physiol. (2008) 294:F1–9. doi: 10.1152/ajprenal.00424.2007, PMID: 17928410

[65] Wever R Boer P Hijmering M Stroes E Verhaar M Kastelein J . Nitric oxide production is reduced in patients with chronic renal failure. Arterioscler Thromb Vasc Biol. (1999) 19:1168–72. doi: 10.1161/01.atv.19.5.1168, PMID: 10323766

[66] Emrich IE Zawada AM Martens-Lobenhoffer J Fliser D Wagenpfeil S Heine GH . Symmetric dimethylarginine (SDMA) outperforms asymmetric dimethylarginine (ADMA) and other methylarginines as predictor of renal and cardiovascular outcome in non-dialysis chronic kidney disease. Clin Res Cardiol. (2018) 107:201–13. doi: 10.1007/s00392-017-1172-4, PMID: 29101459

[67] Piechowicz J Gamian A Chukwu O Polak-Jonkisz D. Nitric oxide synthesis metabolites-as potential markers in chronic kidney disease in children. Curr Issues Mol Biol. (2022) 44:3518–32. doi: 10.3390/cimb44080242, PMID: 36005138 PMC9406431

[68] Lei C Berra L Rezoagli E Yu B Dong H Yu S . Nitric oxide decreases acute kidney injury and stage 3 chronic kidney disease after cardiac surgery. Am J Respir Crit Care Med. (2018) 198:1279–87. doi: 10.1164/rccm.201710-2150OC, PMID: 29932345 PMC6290943

[69] Kim JH Park SH Kim YW Ha JM Bae SS Lee GS . The traditional herbal medicine, Dangkwisoo-san, prevents cerebral ischemic injury through nitric oxide-dependent mechanisms. Evid Based Complement Alternat Med. (2011) 2011:718302. doi: 10.1155/2011/718302, PMID: 21423636 PMC3057556

[70] Song L Zhang W Tang S Luo S Xiong P Liu J . Natural products in traditional Chinese medicine: molecular mechanisms and therapeutic targets of renal fibrosis and state-of-the-art drug delivery systems. Biomed Pharmacother. (2024) 170:116039. doi: 10.1016/j.biopha.2023.116039, PMID: 38157643

[71] Yin YL Zhu ML Wan J Zhang C Pan GP Lu JX . Traditional Chinese medicine xin-mai-jia recouples endothelial nitric oxide synthase to prevent atherosclerosis in vivo. Sci Rep. (2017) 7:43508. doi: 10.1038/srep43508, PMID: 28252100 PMC5333158

[72] Zhu JQ Song WS Hu Z Ye QF Liang YB Kang LY. Traditional Chinese medicine's intervention in endothelial nitric oxide synthase activation and nitric oxide synthesis in cardiovascular system. Chin J Integr Med. (2015) 10:1–9. doi: 10.1007/s11655-015-1964-1, PMID: 25666326

